# In Vivo Evaluation of the Analgesic and Anti-Inflammatory Activity of *Thymus numidicus* Essential Oil

**DOI:** 10.3390/ph18071031

**Published:** 2025-07-11

**Authors:** Ouardia Chaouchi, Velislava Todorova, Stanislava Ivanova, Elizabet Dzhambazova, Farida Fernane, Nacira Daoudi Zerrouki, Lyudmil Peychev, Kremena Saracheva, Michaela Shishmanova-Doseva, Zhivko Peychev

**Affiliations:** 1 Natural Resources Laboratory, Mouloud Mammeri University of Tizi Ouzou, Tizi Ouzou 15000, Algeria; 2 Department of Pharmacognosy and Pharmaceutical Chemistry, Faculty of Pharmacy, Medical University-Plovdiv, 4002 Plovdiv, Bulgaria; 3 Research Institute, Medical University-Plovdiv, 4002 Plovdiv, Bulgaria; 4 Department of Social Medicine and Public Health, Faculty of Public Health, Medical University-Plovdiv, 4002 Plovdiv, Bulgaria; 5 Department of Pharmacology, Toxicology and Pharmacotherapy, Faculty of Pharmacy, Medical University-Plovdiv, 4002 Plovdiv, Bulgaria; 6 Department of Medical Informatics, Biostatistics and E-Learning, Faculty of Public Health, Medical University-Plovdiv, 4002 Plovdiv, Bulgaria

**Keywords:** *Thymus numidicus*, analgesic effect, anti-inflammatory effect, essential oil, volatile constituents

## Abstract

**Background**: *Thymus numidicus* Poiret. (Lamiaceae) is an endemic plant with well-known antibacterial properties. It has been largely used in traditional Algerian medicine. This study aimed to compare the chemical composition of essential oils (EOs) extracted from leaves and flowers using the gas chromatography–mass spectrometry method, as well as to investigate its analgesic and anti-inflammatory activities. **Results**: The EOs were rich in monoterpenes and classified as a thymol chemotype. In vivo experiments revealed that acute treatment with *T. numidicus* EO (20 and 80 mg/kg) significantly increased the thermal threshold on the hot-plate at all tested hours compared to the control animals (*p* < 0.001, respectively), while only the higher dose had a similar effect to the metamizole group at 2 and 3 h. In the mechanical stimulus test, both doses of the EO led to a late analgesic effect presented with increased paw withdrawal threshold only during the third hour compared to the control group (*p* < 0.05, respectively). In the plethysmometer test both doses of the EO dose-dependently reduced paw volume with nearly 10% and 15% compared to the control animals at all tested hours (*p* < 0.001, respectively), with a more pronounced volume reduction in the higher dose. In a neuropathic pain model, the EO (20 mg/kg and 80 mg/kg) dose-dependently increased the withdrawal latency time towards thermal stimuli and enhanced the paw withdrawal threshold in response to mechanical pressure at all tested hours compared to the CCI-group (*p* < 0.001, respectively). These findings demonstrate the potent analgesic and anti-inflammatory effects of *T. numidicus* EO in models of acute and neuropathic pain.

## 1. Introduction

Essential oils (EOs) are volatile, naturally occurring compounds produced by aromatic plants as secondary metabolites [1]. They are complex mixtures comprising over 300 distinct compounds with low molecular weights of less than 300 Daltons. They contain various chemical classes, including alcohols, ethers, oxides, aldehydes, ketones, phenols, heterocycles, and, predominantly, terpenes [1,2,3]. EOs are known to possess antibacterial, antioxidant, anti-inflammatory, cancer chemoprotective, repellent, and insecticidal activities [1,2,3]. Some essential plant families rich in EO are Lamiaceae, Myrtaceae, Asteraceae, etc. [4,5,6,7].

Many aromatic and medicinal plants belonging to the Lamiaceae family are found in the Mediterranean [8]. The Lamiaceae family includes approximately 245 genera. This family is an important source of EOs [9]. One of the largest genera of this family is Thymus. It contains over 215 species and is mainly found in the West Mediterranean area [10]. *Thymus species* are commonly used plants in traditional medicine in various countries. They are used as a spice and preservative [10]. Notably, EOs derived from *Thymus* species are considered highly biologically active due to their high phenolic content, primarily consisting of thymol and carvacrol. Antioxidant and antibacterial capabilities are the foundation of several applications in raw and processed food preservation and pharmaceutical products [11]. A representative of this genus is the endemic perennial, growing wild species *Thymus numidicus* Poiret. [10,12,13]. It is used in traditional medicine to treat colds, flu, respiratory, and digestive diseases and as a spice in food preparations, just like many other *Thymus species*. It has attracted the attention of researchers for its essential oil [10]. Moreover, *T. numidicus* is known for its anti-inflammatory, antiseptic, antibacterial, antifungal, antioxidative, sedative, antispasmodic, and antirheumatic activities [14,15,16,17,18].

The chemotype of the *T. numidicus* EO is thymol [10,19,20]. The chemical composition of EO is very complex. Compounds isolated from EOs include *p*-cymene, carvacrol, linalool, thymol methyl ether, *γ*-terpinene, etc. [21]. Thymol is a monoterpene phenol and possesses therapeutic activities, including antifungal, antioxidant, antibacterial, and anti-inflammatory [16]. Carvacrol has antioxidant, antimicrobial, and anticancer activities [22,23]. Generally, the EO of *T. numidicus* showed intense insecticidal, antifungal, antibacterial, antioxidant, and allelopathic activities [13,21]. In vivo studies, particularly those on the anti-inflammatory and analgesic effects of *T. numidicus* EO, are insufficient.

Inflammation itself is a complex biological response to harmful stimuli such as pathogens, damaged cells, or irritants. It plays a crucial role in eliminating these stimuli and initiating tissue repair. Depending on its duration and progression, inflammation is categorized as acute or chronic [24,25,26]. Although anti-inflammatory medications such as non-steroidal anti-inflammatory drugs and corticosteroids are commonly used, their long-term use is often associated with adverse side effects. As a result, there is growing interest in alternative therapies, including those derived from traditional medicine. Essential oils, in particular, have shown promising therapeutic potential. Notably, many phenylpropanoids and monoterpenes found in EOs have been shown to exert anti-inflammatory effects, making them potential candidates for developing safer, plant-based anti-inflammatory agents [24,27,28].

This study aimed to compare the chemical composition of *T. numidicus* EO from leaves and flowers. Additionally, the antinociceptive and anti-inflammatory effects of *T. numidicus* EO were also evaluated in Wistar rats.

## 2. Results and Discussion

### 2.1. GC-MS Analysis of Isolated EOs

**Figure 1:**
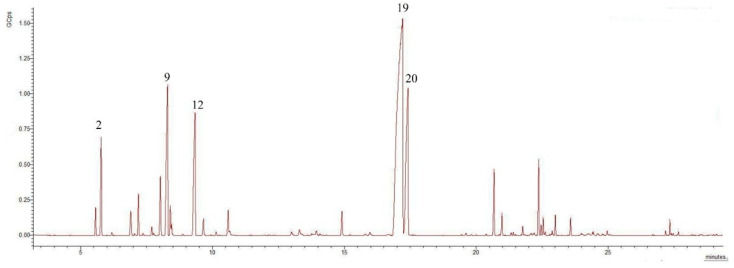
Chromatogram of *T. numidicus* EO from leaves, where 2-*α*-pinene, 9-*p*-cymene, 12-*γ*-terpinene, 19-thymol, 20-carvacrol.

**Figure 2:**
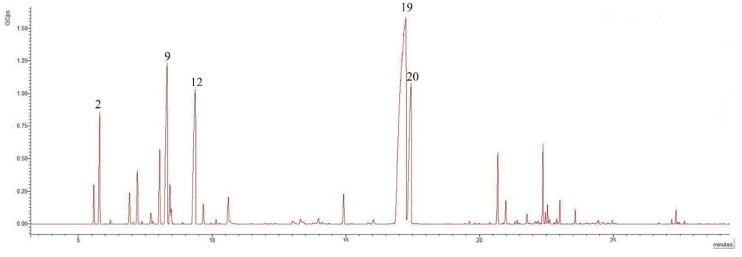
Chromatogram of *T. numidicus* EO from flowers, where 2-*α*-pinene, 9-*p*-cymene, 12-*γ*-terpinene, 19-thymol, 20-carvacrol.

The EO obtained after extraction was an intensely fragrant essential oil with an orange–yellow color, and this is due to the plant’s chemical composition; the presence of carotenoids influences the color of the EO. The extraction yields of leaves and flowers were 2.88% and 3.17%, respectively. The representative chromatograms of *T. numidicus* EO from leaves and flowers are presented in Figure 1 and Figure 2, respectively. The identified chemical components from both EOs are presented in Table 1. Our results correspond with those of several studies conducted on *T. numidicus* EO [13,15,19], and it is preferable that the harvest should be undertaken during the flowering period to increase yield [29].

The result of the GC-MS analysis identified 26 compounds in the EO of leaves and 25 compounds in the EO of flowers. According to the results of the CG-MS analysis, the chemical compositions of the essential oils of the flowers and leaves are approximately identical. The analysis of the EO composition revealed a significant presence of monoterpenes and oxygenated monoterpenes in both leaves and flowers, with flowers exhibiting a higher percentage of monoterpenes (32.13%) compared to leaves (27.68%, *p* = 0.049 *). Oxygenated monoterpenes are more prevalent in leaves (63.53%) than in flowers (59.25%, *p* = 0.021 *). The major compounds identified include thymol and carvacrol, which are present in substantial amounts in both leaves and flowers. Thymol is slightly more prevalent in leaves (47.13%) than in flowers (45.37%, *p* = 0.069), whereas carvacrol accounts for 12.75% and 10.86% in flowers (*p* = 0.004 **). These phenolic compounds are known for their antimicrobial and antioxidant properties, which may contribute to the overall therapeutic potential of EOs. Notably, the concentrations of *p*-cymene (10.10%) and *γ*-terpinene (9.12%) are higher in flowers, suggesting that the floral parts of the plant possess a more complex aromatic profile, potentially enhancing their suitability for applications in aromatherapy and perfumery. Although present in trace amounts, 1-octen-3-ol and 3-octanol contribute to the fragrance. L-terpinen-4-ol is present in the leaves (0.62%), but is absent in the flowers. Sesquiterpenes and oxygenated sesquiterpenes were relatively low, with a combined total of 6.77% in leaves and 6.28% in flowers (*p* = 0.005 *). This indicates that the essential oil predominantly comprises monoterpenes and their oxygenated derivatives. Camphene and endo-borneol: both compounds are found in trace amounts in leaves and flowers. They can influence the fragrance and biological properties of EOs, even in small quantities. *α*-Pinene is 4.09% in flowers and 3.72% in leaves (*p* = 0.188), while (−)-*β*-pinene is 1.78% in flowers and 1.45% in leaves (*p* = 0.03 *). The levels of caryophyllene were relatively similar in leaves (2.41%) and flowers (2.46%, *p* = 0.434), while being slightly more prevalent in leaves, with *β*-bisabolene at 2.46% in leaves and 2.35% in flowers, *p* = 0.244. Several compounds exhibited statistically significant differences in their concentrations between the leaves and flowers. 1-Octen-3-ol was significantly higher in flowers (*p* = 0.001), as were (−)-*β*-pinene (* *p* = 0.030), *α*-phellandrene (* *p* = 0.046), *α*-terpinene (* *p* = 0.036), D-limonene (* *p* = 0.030), *β*-phellandrene (* *p* = 0.046), and *γ*-terpinene (* *p* = 0.015), all contributing to the stronger fragrance of the floral essential oil. In contrast, linalool (** *p* = 0.009), carvacrol (** *p* = 0.004), and caryophyllene oxide (** *p* = 0.005) were significantly more abundant in leaves, reflecting their higher potential for antimicrobial activity. Additionally, thymol methyl ether was slightly more concentrated in flowers (* *p* = 0.023). These statistically significant differences underline the distinct functional roles of each plant organ in essential oil composition. The higher monoterpene content in flowers makes them suitable for perfumery and aromatherapy applications, providing a strong and pleasant fragrance. The high concentration of oxygenated monoterpenes in leaves suggests that monoterpenes may be more effective for medicinal purposes due to their enhanced antimicrobial and antioxidant properties. Overall, the EO composition highlights the rich and diverse profile of bioactive compounds, which may be responsible for their medicinal and aromatic properties. Further studies on the biological activities of these individual compounds are required to provide deeper insights into their potential applications.

Previously, Messara et al. reported that EOs from *Thymus numdicus* harvested in the wild habitat of Tifrit are predominantly composed of monoterpenes, with thymol and carvacrol being the major compounds [21]. Benayache et al. reported that in Constantine, the oxygenated terpenoid fraction was the most significant, with thymol and *p*-cymene being the predominant compounds [14]. Furthermore, Z. Kabouche et al. analyzed the EO of *T. numidicus* cultivated in Constantine, identifying thymol and carvacrol as the major components. The high thymol concentration aligns with findings from other regions, although some variations in the relative proportions were observed [19]. Saidj et al. identified 14 components in the EO from *T. numidicus* collected in Yakouren, Tizi Ouzou. The primary components were thymol, followed by carvacrol and linalool. These results further confirm the significant presence of thymol and carvacrol in *T. numidicus* EOs across different regions, with varying concentrations [30].

Lauer et al. compared the main components of EOs from purplish-white and violet varieties of *T. numidicus*, finding thymol, *γ*-terpinene, *p*-cymene, carvacrol, and *α*-pinene to be the major compounds, respectively [31]. Previously analyzed essential oils from the leaves of *T. numidicus* revealed thymol, carvacrol, and linalool as the major constituents [32]. It was also reported that carvacrol was the major component, and *p*-cymene was also significant in *T. numidicus* from the Bejaia region in Algeria [33].

In conclusion, the chemical composition of *T. numidicus* EOs exhibits considerable variability influenced by geographical location and harvest time. Thymol and carvacrol consistently emerged as the dominant compounds, although their relative concentrations and the presence of other minor components could vary significantly. In different regions of Algeria, such as Tizi Ouzou, Tifrit, and Constantine, thymol typically ranges from 40% to 68%, while carvacrol ranges from 9% to 16%. Furthermore, several factors can qualitatively and quantitatively alter the chemical composition of essential oils, such as the state of growth of the plant, the nature of the soil [34], climate, collection period, and geographic location [20].

### 2.2. Analgesic Effect of T. numidicus EO 20 mg/kg and 80 mg/kg After per os Treatment in the Hot-Plate Test in Naïve Animals

**Figure 3:**
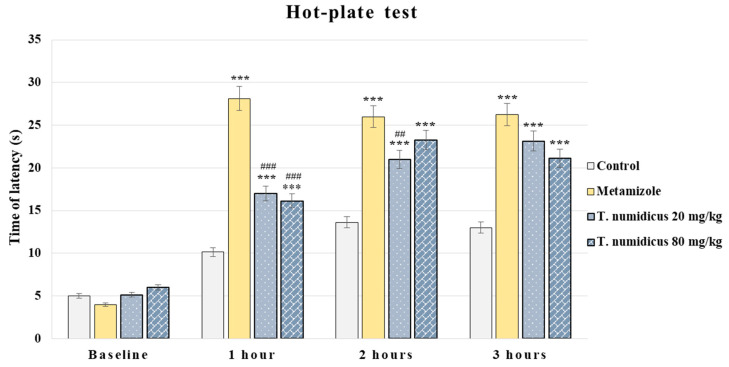
Effect of *T. numidicus* EO 20 mg/kg and 80 mg/kg after per os administration in the hot-plate test in naïve animals. *** *p* < 0.001 compared to the control group; ^##^ *p* < 0.01, ^###^ *p* < 0.001 compared to the metamizole group for the corresponding hour of the experiment.

In the hot-plate test, analysis of the variance revealed an effect of treatment during hour 1 [F(3,28) = 71.422, *p* < 0.001], hour 2 [F(3,28) = 37.673, *p* < 0.001] and hour 3 [F(3,28) = 17.781, *p* < 0.001]. Test of homogeneity of variances indicated no significance and Tukey’s post hoc test was applied for the between-group differences. The reference analgesic drug metamizole demonstrated a significant antinociceptive effect at 1, 2, and 3 h in comparison with the control animals (*p* < 0.001, respectively, Figure 3). The group treated with *T. numidicus* EO 20 mg/kg significantly shortened the time spent on the hot-plate at 1 h (*p* < 0.001), 2 h (*p* < 0.001), and 3 h (*p* = 0.005) compared to the control animals for the corresponding hour, while in comparison with the reference analgesic group treated with metamizole, the EO 20 mg/kg group had less time spent on the plate during the first (*p* < 0.001) and second hours (*p* = 0.002). The 80 mg/kg EO dose of *T. numidicus*, similarly to the lower dose, increased the reaction time during the three testing hours (*p* < 0.001, *p* < 0.001 and *p* = 0.001, respectively) compared to the saline-treated rats. In contrast, the higher dose of 80 mg/kg EO administered at 1 h shortened the time spent on the plate compared to the metamizole-treated animals (*p* < 0.001).

#### 2.2.1. Analgesic Effect of *T*. *numidicus* EO 20 mg/kg and 80 mg/kg After per os Treatment in the Paw Pressure Test (Randall–Selitto Method) in Naïve Animals

**Figure 4:**
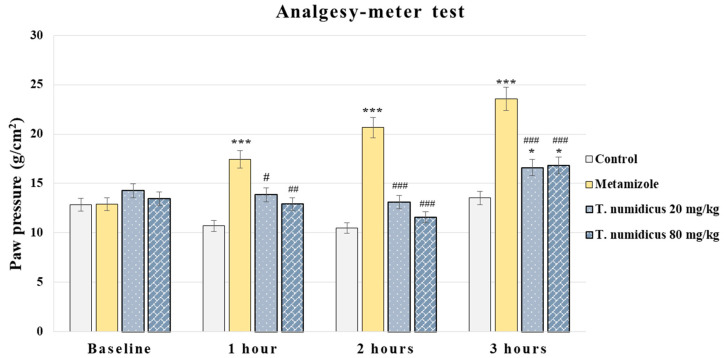
Effect of *T. numidicus* EO 20 mg/kg and 80 mg/kg after per os administration in the paw pressure test in naïve animals. * *p* < 0.05, *** *p* < 0.001 compared to the control group; ^#^ *p* < 0.05, ^##^ *p* < 0.01, ^###^ *p* < 0.001 compared to the metamizole group for the corresponding hour of the experiment.

In the paw pressure test, ANOVA showed a significant effect during hour 1 [F(3,28) = 11.649, *p* < 0.001], hour 2 [F(3,28) = 26.873, *p* < 0.001] and hour 3 [F(3,28) = 34.417, *p* < 0.001]. The Levene’s test was insignificant and Tukey’s post hoc test was applied for the between-group differences. The reference analgesic group treated with metamizole showed a significant analgesic effect at all tested hours compared to the control animals (*p* < 0.001, respectively, Figure 4). Both groups treated with different doses of *T. numidicus* EO (20 and 80 mg/kg) decreased withdrawal latency at 1 h (*p* = 0.023 and *p* = 0.003, respectively), 2 h (*p* < 0.001, respectively), and 3 h (*p* < 0.001, respectively) compared to the metamizole-treated rats. No effect was observed between both groups treated with *T. numidicus* EO, and the control animals at 1 h (*p* = 0.059 and *p* = 0.245, respectively) and 2 h (*p* = 0.183 and *p* = 0.826, respectively). During the third hour, both experimental groups showed a longer reaction time compared to the saline-treated animals (*p* = 0.027 and *p* = 0.016, respectively).

#### 2.2.2. Anti-Inflammatory Effect of *T*. *numidicus* EO 20 mg/kg and 80 mg/kg after per os Treatment in the Plethysmometer Test in Naïve Animals

**Figure 5:**
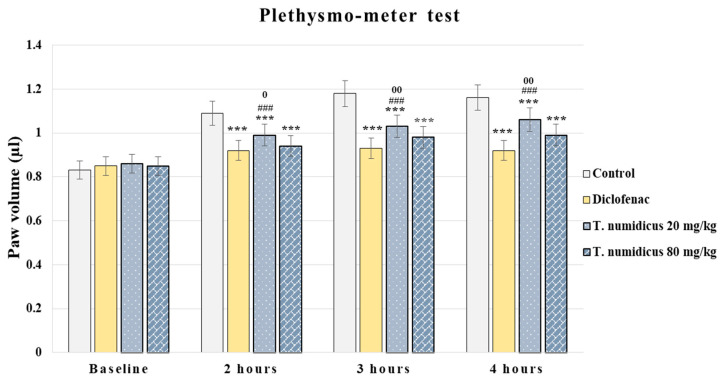
Effect of *T. numidicus* EO 20 mg/kg and 80 mg/kg after each os administration in the plethysmometer test in naïve animals. *** *p* < 0.001 compared to the control group; ^###^ *p* < 0.001 compared to the diclofenac group; ^0^ *p* < 0.05, ^00^ *p* < 0.01 compared to the *T. numidicus* EO 80 mg/kg for the corresponding hour of the experiment.

In the carrageenan model of inflammation, analysis of the variance revealed a treatment effect during hour 2 [F(3,28) = 57.925, *p* < 0.001], hour 3 [F(3,28) = 90.340, *p* < 0.001] and hour 4 [F(3,28) = 67.197, *p* < 0.001]. Test of homogeneity of variances indicated significance for the second hour and Games–Howell post hoc test was applied for the between-group differences, while no significance in the Levene’s test was detected for the third and fourth hours and Tukey’s post hoc test was applied. Diclofenac sodium, used as a reference compound, significantly reduced paw edema at all tested hours compared to the control animals (*p* < 0.001, respectively, Figure 5). *T. numidicus* EO at a dose of 20 mg/kg significantly suppressed carrageenan-induced edema at 2, 3, and 4 h (*p* < 0.001, respectively) compared to the control animals, but the effect was not so pronounced and this group showed greater paw edema in comparison with the diclofenac-treated animals during all tested hours (*p* < 0.001, respectively). In contrast, at a dose of 80 mg/kg *T. numidicus* EO significantly inhibited paw edema during all investigated hours compared to the control rats (*p* < 0.001, respectively), and its effect was similar to that of the diclofenac-treated animals. Moreover, the higher-dose treated group showed a greater effect compared to the low-dose treated animals (20 mg/kg), and a statistical significance was detected between both groups at 2 h (*p* = 0.031), 3 h (*p* = 0.01), and 4 h (*p* = 0.008).

#### 2.2.3. Neuropathic Pain Model Effect of *T. numidicus* EO 20 mg/kg and 80 mg/kg After per os Treatment on CCI-Induced Thermal Hyperalgesia in Rats

**Figure 6:**
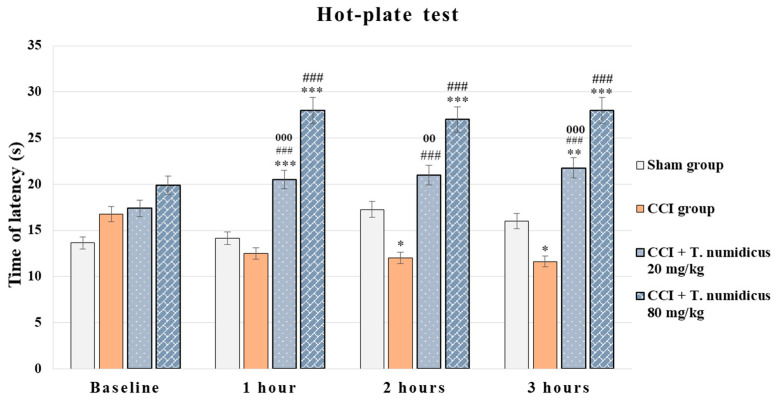
Effect of *T. numidicus* EO 20 mg/kg and 80 mg/kg after per os treatment in the hot-plate test in animals with a model of neuropathic pain (CCI). * *p* < 0.05, ** *p* < 0.01, *** *p* < 0.001 compared to the sham control group; ^###^ *p* < 0.001 compared to the CCI group; ^00^ *p* < 0.01, ^000^ *p* < 0.001 compared to the CCI + *T. numidicus* EO 80 mg/kg for the corresponding hour of the experiment.

ANOVA demonstrated a significant treatment effect during hour 1 [F(3,28) = 70.061, *p* < 0.001], hour 2 [F(3,28) = 26.955, *p* < 0.001] and hour 3 [F(3,28) = 48.053, *p* < 0.001]. The Levene’s test was insignificant and Tukey’s post hoc test was applied for the between-group differences. The group with a model of neuropathic pain (CCI group) had a significantly shorter hot-plate latency time at 2 h (*p* = 0.024) and 3 h (*p* = 0.026) compared to the sham control animals on day 7 after the surgery (Figure 6). Both groups treated with 20 mg/kg and 80 mg/kg EO of *Thymus numidicus* significantly increased the reaction time at 1 h (*p* < 0.001, respectively), 2 h (*p* < 0.001, respectively), and 3 h (*p* < 0.001, respectively) compared to the model group. In addition, the animals treated with the higher dose of the EO significantly lengthened the time spent on the plate at the first (*p* < 0.001), second (*p* = 0.008), and third (*p* = 0.001) hours in comparison with the group treated with the lower dose of 20 mg/kg.

#### 2.2.4. Effect of *T. numidicus* EO 20 mg/kg and 80 mg/kg after per os Treatment on CCI-Induced Mechanical Allodynia in Rats

**Figure 7:**
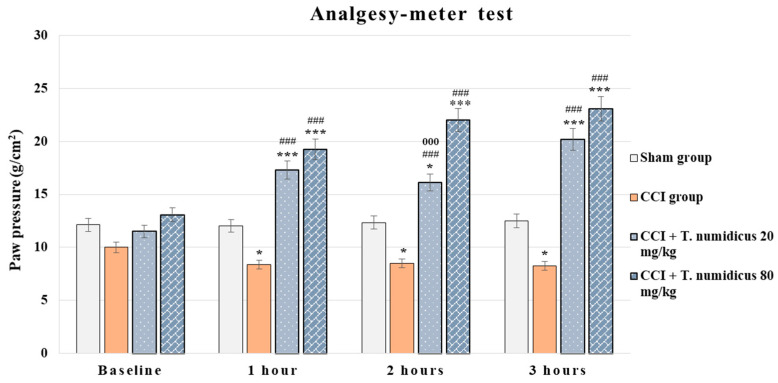
Effect of *T. numidicus* EO 20 mg/kg and 80 mg/kg after per os treatment in the paw pressure test in animals with a model of neuropathic pain (CCI).* *p* < 0.05, *** *p* < 0.001 compared to the sham control group; ^###^ *p* < 0.001 compared to the CCI group; ^000^ *p* < 0.001 compared to the CCI + *T. numidicus* EO 80 mg/kg for the corresponding hour of the experiment.

In the paw pressure test, analysis of the variance revealed a treatment effect during hour 1 [F(3,28) = 57.925, *p* < 0.001], hour 2 [F(3,28) = 90.340, *p* < 0.001] and hour 3 [F(3,28) = 67.197, *p* < 0.001]. The Levene’s test was insignificant for the first and second hours and Tukey’s post hoc test was applied for the between-group differences, while significance in this test was detected only for the third hour and the Games–Howell post hoc test was applied. The model group had a significantly lower reaction time at 1 h (*p* = 0.022), 2 h (*p* = 0.036), and 3 h (*p* = 0.014) than the control animals (Figure 7). At a dose of 20 mg/kg EO of *Thymus numidicus*, latency was significantly increased at all tested hours (*p* < 0.001, respectively) compared with the animals with a model of neuropathic pain. The same effect was observed in the low-dose EO group and the sham group at 1 h (*p* = 0.001), 2 h (*p* = 0.038), and 3 h (*p* < 0.001). The animals treated with the higher dose of 80 mg/kg EO significantly increased the latency of the pressure reaction during the three experimental hours compared with the CCI and sham groups (*p* < 0.001, respectively). Moreover, the *T. numidicus* 80 mg/kg group had a significantly longer time for paw withdrawal compared to the low-dose treatment group at 2 h (*p* = 0.001).

Additional tables about statistical analysis are presented in Tables S1–S5. The results obtained from the present study revealed that *T. numidicus* EO exerted an antinociceptive effect in both acute and neuropathic pain. In the acute experiment, the EO showed greater activity against thermal nociception than mechanical allodynia. In the hot-plate test both applied doses of *T. numidicus* EO demonstrated a significant analgesic effect with increased latency time for paw licking during all tested hours. Moreover, the higher dose of (80 mg/kg) EO produced marked antinociceptive effect with an increased thermal threshold similar to those of the reference drug metamizole. In the mechanical stimulus test, both doses of the EO led to a late analgesic effect presented with an increased paw withdrawal threshold.

To the best of our knowledge, there is no data on the analgesic properties of *T. numidicus*. Recent in vitro and in vivo studies have revealed that *Thymus numidicus* EOs have antifungal and antiproliferative activity as well as protective effects on the kidneys and liver in a model of deltamethrin-induced hematological and biochemical toxicity [35,36]. However, the observed effects in the current study can be attributed to its high content of phenolic compounds, primarily thymol and carvacrol. Our results are in agreement with other studies that have revealed that carvacrol-rich essential oils exert antinociceptive activity by blocking peripheral nerve excitability [37]. This effect is associated with two possible mechanisms: inhibition of glutamatergic neurotransmission, and blockage of the voltage-gated Na^+^-channels, which are responsible for the initiation and prolongation of the pain response [37,38]. Additionally, the analgesic effect of carvacrol is also related to its scavenger activity on different Reactive Oxygen Species and nitric oxide [39]. After local application or intradermal injection of carvacrol, Klein et al. reported that the phenolic compound induces a concentration-dependent increase in thermal withdrawal latency with no significant effect on mechano-sensitivity, which complies with our results [40].

Thymol is the other highly presented phenolic compound in our EO and is widely known for its great variety of pharmacological properties, from which its analgesic activity is highlighted. This effect has been related to several mechanisms, such as partial blockage of voltage-gated Na^+^-channels and direct activation of Cl^−^-currents through GABA_A_ receptors [41]. Moreover, there is data that thymol reversibly inhibits prostaglandin synthesis [42].

Carrageenan-induced hind paw edema is among the tests widely used to screen anti-inflammatory compounds [43]. This model of inflammation has biphasic effects. The early hyperemia, which occurs between 0 and 3 h after carrageenan injection, is related to the release of serotonin, histamine, and bradykinin, whereas the delayed phase, which occurs between 3 and 6 h after carrageenan injection, is associated with prostaglandin release [43,44]. In our study, we found that *T. numidicus* EO significantly reduced inflammation at both applied doses. Moreover, the higher dose of the EO exhibited a more potent decrease in rat paw edema volume, and its results were comparable with those of the reference drug diclofenac during both phases. We observed a dose-dependent effect with an additional reduction in paw edema of almost 5% at all time points in the rats treated with the higher dose of 80 mg/kg EO compared to the animals treated with the lower dose of the EO. Recent data have revealed that carvacrol could decrease inflammation and vascular permeability via different mechanisms, including inhibition of cyclooxygenase −2 expression [45] and decreased production of some pro-inflammatory cytokines such as tumor necrosis factor-*α* (TNF-*α*), interleukin-1*β* (IL-1*β*), and interleukin-6 (IL-6) [46,47,48].

The antinociceptive and anti-inflammatory effects observed in our study could be related to some other highly presented components of the EO, such as *p*-cymene and *γ*-terpinene. The literature data show that *p*-cymene induces considerable analgesic and anti-inflammatory responses in an animal model of cancer-associated pain [49].

The model of chronic constriction injury of the sciatic nerve is associated with thermal, mechanical, and chemical hyperalgesia, as well as cold allodynia [50], which is in line with our current results. We found that the neuropathic-model group decreased thermal withdrawal latency and reduced the paw pressure threshold. In contrast, treatment with different doses of *T. numidicus* EO demonstrated a significant antinociceptive effect in both hot-plate and analgesy-meter tests. Both applied doses (20 mg/kg and 80 mg/kg) of the EO increased the withdrawal latency time towards thermal stimuli and enhanced the paw withdrawal threshold in response to mechanical pressure. Additionally, we observed a dose-dependent effect, and the higher dose of 80 mg/kg EO led to a more pronounced reduction of thermal hyperalgesia during all tested hours and better analgesic effect only during the second hour in the paw pressure test.

Our results are in agreement with recent studies where carvacrol-rich compounds alleviate the hyperalgesia associated with neuropathic pain after spinal cord injury and the CCI-model via different mechanisms including opioidergic pathways [40]; this includes blockage of transient receptor melastatin 7, followed by downregulation of the production of pain-related factors such as IL-1*β*, IL-6, TNF-*α*, and matrix metalloproteinase 9 [51].

Recent data shows that *γ*-terpinene, another highly presented component in our EO, reverses mechanical allodynia and thermal hyperalgesia in an experimental neuropathic model of CCI with possible cannabinoid receptor involvement [52]. Moreover, Pina et al. demonstrate that *γ*-terpinene attenuates spinal neuroactivity and inflammation in animals with cancer by blocking voltage-dependent calcium^2+^-channels.

## 3. Materials and Methods

### 3.1. Chemicals and Reagents

The following hydrocarbons were used to determine the retention indices (RIs): nonane (99%), decane (≥99%), undecane (≥99%), dodecane (99%), tridecane (≥99%), tetradecane (≥99%), hexadecane (≥99%), and heptadecane (99%). These hydrocarbons were purchased from Merck KGaA, Darmstadt, Germany). Hexane GC-grade (Thermo Fisher Scientific GmbH, Bremen, Germany) was used to dilute the EOs. Drugs and compounds were purchased from pharmacy as follows: diclofenac (Diclac, Hexal AG, Holzkirchen, Germany), metamizole (Metamizole Sodium, Sopharma, Sofia, Bulgaria), carrageenan (Honeywell Fluka, Fisher Scientific, Loughborough, UK), and pentobarbital sodium (Merck KGaA, Darmstadt, Germany).

### 3.2. Plant Material and Oil Extraction

In June and July 2022, *Thymus numidicus* Poiret arial parts, leaves and flowers, were harvested during the entire flowering stage at Alma Guechtoum, located at an altitude of 765 m (36°47′57″ N, 4°25′14″ E), near the Tifrit locality in the Tizi Ouzou Province of Algeria. It was identified at the National Institute of Agronomic Research in Algeria and deposited in the herbarium of the National School of Agronomy in El-Harrach, Algeria, under the reference number TN/2022/NSA/50.

The collected plant material was dried outdoors in the shade at room temperature. After a two-week drying period, the essential oil was extracted by steam distillation. One liter of water was used to distil 100 g of dry matter for 2 h. The resulting essential oil was dried with Na_2_SO_4_ and then stored in dark glass vials at 4 °C until use.

### 3.3. Chromatographic Conditions

The gas chromatography–mass spectrometry (GC-MS) analysis was performed on a Bruker Scion 436-GC SQ MS system (Bremen, Germany) fitted with a capillary Zebron ZB-5MSplus column (30 m × 0.25 mm, 0.25 μm film thickness, Torrance, CA, USA). Prior to use, the EOs were diluted with hexane at a ratio of 1:50 (*v*/*v*). The ionization voltage for the mass was 70 eV, and the spectral range was 50–300 m/z in full-scan mode. Initially, the oven temperature was maintained at 60 °C for 1 min, then raised to 130 °C at a rate of 4 °C/min. Finally, the temperature was raised to 220 °C at a rate of 10 °C/min and maintained for 2 min. The carrier gas was helium with a flow rate of 1.0 mL/min; the temperatures of the detector and injector were adjusted to 300 and 250 °C, respectively. The injection volume was 1 μL, and the split mode was 1:20. The molecules were identified based on their retention indices, which were calculated using a series of n-alkanes that had undergone analysis under the same conditions as the sample. The retention indices were then compared to the Wiley NIST 11 mass spectral library, the literature data from ADAMS, and mass spectral characteristic and fragmentations of each compound [53,54].

Statistical analysis of the obtained results:

Statistical analyses were performed in Systat Sigma Plot v15 software (SYSTAT, San Jose, CA, USA), and the data were calculated as mean ± SEM. Variations between the experimental groups were calculated by *t*-test. The level of statistical significance was set at * *p* < 0.05, ** *p* < 0.01 and *** *p* < 0.001. The experimental data presented are representative of three independent experiments.

### 3.4. In Vivo Experiments

#### 3.4.1. Animals

Permission to use animals in the experiment was obtained from the Food Safety Agency of the Bulgarian Ministry of Agriculture and Food (No. 396/23.05.2024, valid until 31 May 2029). The study was formally approved by the Ethical Committee on Human and Animal Experimentation of the Medical University of Plovdiv. The European Community Council directives conducted all procedures: 86/609/EEC.

Male Wistar rats weighing 170–220 g. were used. The animals were kept under standard laboratory conditions: 12 h:12 h dark–light cycle, 45% relative humidity, temperature at 23 ± 1 °C and free access to food and water. In the following experiments for anti-inflammatory and analgesic effects, as well as in the neuropathic pain model, all rats were randomly allocated into four groups, each containing eight animals. The experiments were blinded to the treatments given to the animals, performed behavioral experiments and data analysis.

#### 3.4.2. Experimental Groups

Study of the analgesic effect after treatment with *T. numidicus* EO per os in naïve animals:

To investigate the analgesic activity of *T. numidicus* essential oil, experiments with naïve rats were performed using two methods for inducing nociceptive stimuli: a hot-plate test with thermal pain stimulus and an analgesy-meter causing mechanical pain pressure.

In these experiments, male white Wistar rats were used, randomly divided into four groups of eight animals as follows:

○ 1st group Control-Saline 1 mL/kg b.w. per os;
○ 2nd group-metamizole, 150 mg/kg b.w. i. p.—standard for a drug with analgesic effect;
○ 3rd group-*T. numidicus* EO 20 mg/kg b.w. per os;
○ 4th group-*T. numidicus* EO 80 mg/kg b.w. per os.

Study on the anti-inflammatory effect after treatment with *Thymus numidicus* EO per os in naïve animals:

We used male white Wistar rats, randomly divided into 4 groups of 8 animals as follows:

○ 1st group Control-Saline 1 mL/kg b.w. per os;
○ 2nd group-diclofenac, 25 mg/kg b.w. i. p., positive control—standard for a drug with anti-inflammatory effect;
○ 3d group-*T. numidicus* EO, 20 mg/kg b.w. per os;
○ 4th group-*T. numidicus* EO, 80 mg/kg b.w. per os.

Study of analgesic effects after treatment with *T. numidicus* EO per os in animals with a model of neuropathic pain:

Experiments with rats that had chronic constriction injury (CCI) of the sciatic nerve to study analgesic activity—a model of neuropathic pain [55]:

○ 1st group sham control-operated animals without ligation of the sciatic nerve, which were administered saline solution 1 mL/kg b.w. per os;
○ 2nd group CCI group-operated animals with ligation of sciatic nerve, which were administered saline solution 1 mL/kg b.w. per os;
○ 3rd group-operated animals with ligation of sciatic nerve, which were administered *T. numidicus* EO in a dose of 20 mg/kg b.w. per os;
○ 4th group-operated animals with ligation of sciatic nerve, which were administered *T. numidicus* EO in a dose of 80 mg/kg b.w. per os.

#### 3.4.3. Experimental Methods

##### Hot-Plate Test for Analgesic Activity

A HOT/COLD PLATE apparatus (Ugo Basile, Gemonio, Italy) was used with a hot surface at a temperature of 55 ± 50 °C. The animals were placed on it, confined by a Plexiglas cylinder. The latency time in seconds was recorded, defined as the period between the moment the animal was placed on the hot-plate and the moment it licked one of its hind paws, attempted to jump out of the cylinder, or responded with vocalization. To prevent tissue damage, the maximum time spent on the plate (cut-off time) was 30 s. The latency time was recorded for each animal immediately after treatment, as well as at the first, second, and third hour after treatment. The criterion for analgesic activity was the prolongation of the normal reaction time in the experimental animals compared with the control animals treated with saline. The metamizole-treated control group was used as a reference for assessing the analgesic effect.

##### Nociceptive Test Using Mechanical Pressure to Assess the Analgesic Effect

The test was described by Randall and Selitto [56]. An analgesy-meter (Ugo Basile, Gemonio, Italy) was used. A mechanical pain stimulus was applied. The pain threshold was measured by applying pressure to one of the hind paws of the rat. The pressure was calibrated at 10 g/cm with a maximum force of 250 g. The pressure at which the animal withdraws the tested paw was reported in conventional units (centimeters). The maximum possible pressure is 25 cm. Testing is performed for each animal immediately after treatment, as well as at the first, second, and third hour.

##### Neuropathic Pain Test to Investigate the Analgesic Effect

A model of neuropathic pain was created by ligation of the sciatic nerve [55]. Animals are anesthetized via intraperitoneal administration of Pentobarbital sodium at a dose of 50 mg/kg b.w., which provides anesthesia lasting 20–40 min. Animals are quickly put to sleep and fixed on their stomachs on a board. The upper third of the left leg is shaved with an electric razor, and the area is disinfected with alcohol. A skin incision of approximately 2 cm is made. The subcutaneous tissue was carefully dissected using blunt scissors and was lightly cut. The left sciatic nerve is located deep between the biceps femoris muscle. The nerve is gently dissected from the surrounding tissues, and two ligatures (each with two knots) are made 2 mm apart. The first ligature is looser than the second, but it compresses the nerve without cutting it. After nerve damage, the subcutaneous tissue and skin are sutured with surgical sutures. The wound is disinfected with sulphathiazole powder. In the sham control, an identical dissection of the left leg is performed without ligating the sciatic nerve. Seven days after the ligation of the sciatic nerve, the experiments on the analgesic effect of *T*. *numidicus* EO are evaluated.

##### Method for Testing Anti-Inflammatory Activity

To study the anti-inflammatory effect of *T. numidicus* EO, 0.1 mL of a 1% solution of carrageenan in saline was injected into the right hind paw of a rat, which caused paw edema.

Using a plethysmometer (Ugo Basile, Gemonio, Italy), the initial volume in milliliters of the hind right paw of the animals before carrageenan injection (Start time) was measured. Then, 0.1 mL of a 1% solution of carrageenan in saline was injected into the hind right paw of all animals to cause carrageenan edema. Immediately after the administration of carrageenan, the animals from the 1st Control group were injected i.p. with 0.1 mL/kg b.w. saline; the second group was administered diclofenac at a dose of 25 mg/kg b.w. i.p., and the animals from the third and fourth groups received *T. numidicus* EO at doses of 20 and 80 mg/kg b.w. per os, respectively.

The magnitude of paw edema was determined using a plethysmometer (Ugo Basile, Gemonio, Italy) by measuring the volume of water displaced in mL when the paw was immersed in the device. The first measurement (Start time) was performed immediately before the injection of carrageenan. After the injection of carrageenan, measurements of the volume of displaced fluid from the right hind paw of the rat were made at the first, second, and third hour. The difference in mL between the volume of the carrageenan-treated right hind paw at the second, third, and fourth hour (*V_t_*) and the volume (*V*_0_) of the right hind paw of each experimental animal before treatment was calculated. The percentage of inflammation elimination was calculated using the following Trinus formula:

(1) $$Paw\;swelling\;(\%)=\frac{V_{t}-V_{0}}{V_{0}}\times 100$$

A marker for anti-inflammatory effect is a reduction in paw swelling.

Statistical analysis of the obtained results.

The data obtained from the nociceptive and anti-inflammatory tests were analyzed using parametric tests because of normally distributed data, as assessed by the Kolmogorov–Smirnov test. All different parameters, including latency time, paw pressure and paw volume, were analyzed by one-way ANOVA. When the F-ratio was not significant, depending on the homogeneity of the dispersions (found by using the Levene’s test), the between-group differences were assessed by Tukey’s post hoc test in case of justification. When the variances were significantly different, then the Games–Howell post hoc test was applied. Statistical significance was set at *p* < 0.05. The analysis was conducted by using the IBM SPSS^®^ (version 19.0.) statistical package.

A limitation of the present study is the absence of direct measurement of levels of pro-inflammatory cytokines, which could better support the observed anti-inflammatory results. Moreover, chronic toxicity assessment, sex differences and pharmacokinetic parameters would provide more precise information about the effect of the EO and its safety in humans. All these topics are planned in our future research.

## 4. Conclusions

The essential oil from *Thymus numidicus* has several small but notable chemical differences between those isolated from leaves and flowers. The essential oil studies from the Tizi Ouzou Province of Algeria showed that thymol (leaves: 47.13% and flowers: 45.37%) was present in the highest percentage, followed by carvacrol. Other significant components include *p*-cymene, γ-terpinene, *α*-pinene and *β*-pinene. Furthermore, factors such as plant growth stage, soil type, and climate play a crucial role in determining the EO’s composition. In addition, the obtained in vivo experiments demonstrated the pronounced antinociceptive effects of *T. numidicus* EO, especially against thermal nociception than mechanical allodynia in naïve animals. *Thymus numidicus* EO produced a marked anti-inflammatory effect in a dose-dependent manner. Moreover, in the neuropathic model of chronic constriction injury, treatment with different doses of *T. numidicus* EO relieved both thermal and mechanical hyperalgesia, with more potent activity at higher doses.

## Figures and Tables

**Table 1:** The GC-MS data for essential oil components were identified in *T. numidicus* leaves and flowers, where tr was traced, with a level of less than 0.2%, where MH-Monoterpene hydrocarbons, MO-Oxygenated monoterpene, SH-Sesquiterpene hydrocarbons, SO-Oxygenated sesquiterpene, O-Others tr: trace, Ric-retention index calculated, Ril-retention index literature Percentages are presented as the mean of three independent experiments; standard deviations did not exceed 2% and are omitted for better visualization of the results. Statistical differences between leaf and flower compositions were evaluated using a t-test with * *p* < 0.05, ** *p* < 0.01 and *** *p* < 0.01.

No.	Compounds	Formula	Class	RIc	RIl	% of Total-Leaves	% of Total-Flowers	*p*-Value
1	*α*-Thujene	C_10_H_16_	MH	929	924	0.86	1.04	0.078
2	*α*-Pinene	C_10_H_16_	MH	933	936	3.72	4.09	0.188
3	Camphene	C_10_H_16_	MH	950	950	tr	tr	1.000
4	1-Octen-3-ol	C_8_H_16_O	O	977	980	1.05	1.13	0.001 ***
5	(−)-*β*-Pinene	C_10_H_16_	MH	982	984	1.45	1.78	0.030 *
6	3-Octanol	C_8_H1_8_O	O	987	988	tr	ND	0.005 **
7	*α*-Phellandrene	C_8_H_16_	MH	1009	1008	0.29	0.34	0.046 *
8	*α*-Terpinene	C_10_H_16_	MH	1017	1015	2.41	2.83	0.036 *
9	*p*-Cymene	C_10_H_14_	MH	1019	1020	9.12	10.10	0.067
10	D-Limonene	C_10_H_16_	MH	1020	1024	1.03	1.83	0.030 *
11	*β*-Phellandrene	C_10_H_16_	MH	1022	1025	0.37	0.42	0.046 *
12	*γ*-Terpinene	C_10_H_16_	MH	1050	1057	8.43	9.70	0.015 *
13	trans-*β*-Terpineol	C_10_H_18_O	MO	1061	1159	0.67	0.70	0.194
14	Linalool	C_10_H_18_O	MO	1090	1099	1.06	0.96	0.009 **
15	Endoborneol	C_10_H_18_O	MO	1162	1165	tr	tr	1.000
16	L-terpinen-4-ol	C_10_H_18_O	MO	1176	1179	0.62	tr	0.122
17	L-*α*-Terpineol	C_10_H_18_O	MO	1190	1186	0.30	0.31	0.614
18	Thymol methyl ether	C_11_H_16_O	MO	1229	1237	1.00	1.05	0.023 *
19	Thymol	C_10_H_14_O	MO	1298	1301	47.13	45.37	0.069
20	Carvacrol	C_10_H_14_O	MO	1300	1298	12.75	10.86	0.004 **
21	Caryophyllene	C_15_H_24_	SH	1414	1417	2.41	2.46	0.434
22	*γ*-Muurolene	C_15_H_24_	SH	1471	1476	0.26	0.37	0.100
23	*β*-Bisabolene	C_15_H_24_	SH	1486	1481	2.46	2.35	0.244
24	*γ*-Cadinene	C_15_H_24_	SH	1510	1513	0.54	0.29	0.904
25	*δ*-Cadinene	C_15_H_24_	SH	1519	1522	0.57	0.45	0.380
26	Caryophyllene oxide	C_15_H_24_O	SO	1576	1578	0.50	0.36	0.005 **
			Monoterpene hydrocarbons			27.68	32.13	0.049 *
			Oxygenated monoterpene			63.53	59.25	0.021 *
			Sesquiterpene hydrocarbons			6.24	5.92	0.417
			Oxygenated sesquiterpene			0.50	0.36	0.005 **
			Others			1.05	1.13	0.028 *
			Total			99.00	98.79	0.677

## Data Availability

Data is contained within the article or Supplementary Materials.

## Supplementary Materials

The following supporting information can be downloaded at: https://www.mdpi.com/article/10.3390/ph18071031/s1, Table S1. Summary data of Hot-plate test on mean values ± standard error of mean (SEM) in naïve animals, treated with metamizole and *T. numidicus* EO 20 mg/kg and 80 mg/kg. Table S2. Summary data of Paw pressure test on mean values ± standard error of mean (SEM) in naïve animals, treated with metamizole and *T. numidicus* EO 20 mg/kg and 80 mg/kg. Table S3. Summary data of Plethysmometer test on mean values ± standard error of mean (SEM) in naïve animals, treated with diclofenac and *T. numidicus* EO 20 mg/kg and 80 mg/kg. Table S4. Summary data of Hot-plate test on mean values ± standard error of mean (SEM) in animals with a model of neuropathic pain (CCI), treated with *T. numidicus* EO 20 mg/kg and 80 mg/kg. Table S5. Summary data of Paw pressure test on mean values ± standard error of mean (SEM) in animals with a model of neuropathic pain (CCI), treated with *T. numidicus* EO 20 mg/kg and 80 mg/kg.

## Abbreviations

The following abbreviations are used in this manuscript:

EO	Essential oil
MH	Monoterpene hydrocarbons
MO	Oxygenated monoterpene
SH	Sesquiterpene hydrocarbons
SO	Oxygenated sesquiterpene
O	Others
tr	traces
RIc	retention index calculated
RIl	retention index literature
