# Supplementary material for: In Vivo Evaluation of the Analgesic and Anti-Inflammatory Activity of Thymus numidicus Essential Oil
Source: Pharmaceuticals (Basel). 2025 Jul 11;18(7):1031. doi: 10.3390/ph18071031 (PMC12300282; doi:10.3390/ph18071031)
Supplement: Supplementary file 1 [file pharmaceuticals-18-01031-s001.zip › pharmaceuticals-3697331-supplementary.pdf]

SUPPORTING INFORMATION

Table S1. Summary data of Hot-plate test on mean values ± standard error of mean (SEM) in naïve animals, treated with Metamizole and *T. numidicus* EO 20 mg/kg and 80 mg/kg.

Groups	Baseline Mean ± SEM	1 hour Mean ± SEM	2 hours Mean ± SEM	3 hours Mean ± SEM
Control	5.00 ± 0.33	10.13 ± 1.16	13.63 ± 0.78	13.00 ± 0.78
Metamizole	4.00 ± 0.42	28.13 ± 0.99	26.00 ± 0.87	26.25 ± 1.01
T. numidicus 20	5.13 ± 0.61	17.00 ± 0.76	21.00 ± 1.10	23.13 ± 1.12
T. numidicus 80	6.00 ± 0.54	16.13 ± 0.52	23.25 ± 0.65	23.13 ± 1.16

Table S2. Summary data of Paw pressure test on mean values ± standard error of mean (SEM) in naïve animals, treated with Metamizole and *T. numidicus* EO 20 mg/kg and 80 mg/kg.

Groups	Baseline Mean ± SEM	1 hour Mean ± SEM	2 hours Mean ± SEM	3 hours Mean ± SEM
Control	12.84 ± 0.69	10.70 ± 0.84	10.50 ± 0.79	13.55 ± 0.42
Metamizole	12.90 ± 0.63	17.44 ± 0.87	20.66 ± 0.99	23.54 ± 0.42
T. numidicus 20	14.28 ± 0.48	13.86 ± 0.78	13.11 ± 0.69	16.60 ± 0.87
***T. numidicus* 80**	**13.45 ± 0.98**	**12.93 ± 0.79**	**11.58 ± 0.81**	**16.83 ± 0.97**

Table S3. Summary data of Plethysmometer test on mean values ± standard error of mean (SEM) in naïve animals, treated with Diclofenac and *T. numidicus* EO 20 mg/kg and 80 mg/kg.

Groups	Baseline Mean ± SEM	2 hours Mean ± SEM	3 hours Mean ± SEM	4 hours Mean ± SEM
Control	0.83 ± 0.005	1.09 ± 0.017	1.18 ± 0.008	1.16 ± 0.008
Diclofenac	0.85 ± 0.004	0.92 ± 0.008	0.93 ± 0.005	0.91 ± 0.016
T. numidicus 20	0.86 ± 0.004	0.99 ± 0.007	1.03 ± 0.008	1.06 ± 0.010
T. numidicus 80	0.85 ± 0.006	0.94 ± 0.005	0.97 ± 0.009	0.99 ± 0.014

Table S4. Summary data of Hot-plate test on mean values ± standard error of mean (SEM) in animals with a model of neuropathic pain (CCI), treated with *T. numidicus* EO 20 mg/kg and 80 mg/kg.

Groups	Baseline Mean ± SEM	1 hour Mean ± SEM	2 hours Mean ± SEM	3 hours Mean ± SEM
Sham group	13.63 ± 1.13	14.13 ± 0.89	17.25 ± 1.04	16.00 ± 0.68
CCI group	16.75 ± 0.65	12.50 ± 0.91	12.00 ± 1.01	11.63 ± 0.59
CCI+*T. numidicus* 20	17.38 ± 0.52	20.50 ± 0.71	21.00 ± 1.23	21.75 ± 0.71
CCI+*T. numidicus* 80	19.88 ± 0.98	28.00 ± 0.57	27.00 ± 1.13	28.00 ± 0.54

Table S5. Summary data of Paw pressure test on mean values ± standard error of mean (SEM) in animals with a model of neuropathic pain (CCI), treated with *T. numidicus* EO 20 mg/kg and 80 mg/kg.

Groups	Baseline Mean ± SEM	1 hour Mean ± SEM	2 hours Mean ± SEM	3 hours Mean ± SEM
Sham group	12.13 ± 0.76	12.04 ± 0.71	12.33 ± 0.92	12.50 ± 0.98
CCI group	10.00 ± 0.86	8.38 ± 0.77	8.50 ± 0.66	8.25 ± 0.68
CCI+*T. numidicus* 20	11.50 ± 0.85	17.29 ± 0.70	16.33 ± 0.99	20.19 ± 0.82
CCI+*T. numidicus* 80	13.08 ± 0.74	19.25 ± 0.98	22.00 ± 0.97	23.08 ± 0.77
